# Gravity center estimation for evaluation of standing whole body compensation using virtual barycentremetry based on biplanar slot-scanning stereoradiography - validation by simultaneous force plate measurement

**DOI:** 10.1186/s12891-021-04948-5

**Published:** 2022-01-03

**Authors:** Kazuhiro Hasegawa, Celia Amabile, Matthieu Nesme, Jean Dubousset

**Affiliations:** 1 Niigata Spine Surgery Center, 2-5-22 Nishi-machi, Niigata City, 950-0165 Japan; 2 EOS imaging, 10 rue Mercoeur, 75011 Paris, France; 3 AnatoScope, Montpellier Headquarter, Cap-Omega Rond-Point Benjamin ranklin, 34960 Montpellier, France; 4 Académie Nationale de Médecine, 16 Rue Bonaparte, 75006 Paris, France

**Keywords:** Barycentremetry, EOS®system, Force plate measurement, Gravity center, Whole body standing alignment

## Abstract

**Background:**

Whole body standing alignment (WBSA) in terms of biomechanics can be evaluated accurately only by referring the gravity line (GL) which lies on the gravity center (GC). Here, we introduce a method for estimating GL and simultaneous WBSA measurement using the EOS® imaging system and report on the reproducibility and reliability of the method.

**Methods:**

A 3-dimensional (3D) avatar to estimate GC was created following three steps: 3D reconstruction of the bone based on EOS images; deformation into a generic morphotype (MakeHuman statistical model) before density integration with 3D rasterization of the full body into 1-mm^3^ voxels (the content of each voxel is considered homogeneous); computation of the density of all the voxels provides the center of mass, which can be projected onto the floor as the GC of the full body, providing the GL in relation to the WBSA. The repeatability, reproducibility, and accuracy of the estimated GC and body weight of the avatar were compared with clinical estimation using a force plate in healthy volunteers and patients with degenerative and deformative diseases.

**Results:**

Statistical analyses of the data revealed that the repeatability and reproducibility of the estimation was high with intra-rater and inter-rater intraclass correlation coefficient. ≥0.999. The coordinate values of the GC and body weight estimation did not differ significantly between the avatar and force plate measurements, demonstrating the high accuracy of the method.

**Conclusion:**

This new method of estimating GC and WBSA is reliable and accurate. Application of this method could allow clinicians to quickly and qualitatively evaluate WBSA with GL with various spinal malalignment pathologies.

## Background

Humans stand with the chain of balance beginning at the feet, progressing to the lower limb joints (i.e., ankles, knees, hip joints, and pelvis), the spinal segments, and finally to the cranium, which acts as a pendulum to achieve horizontal vision. The skeletal elements work in concert to maintain an erect posture for which the “cone of economy” represents perfect balance requiring minimal muscle activity in normal situations [1]. Aging or progressive spondylosis, however, induces stooping. The resulting sagittal malalignment is compensated by all the parts of the axial skeleton, especially by increases in cervical lordosis, pelvic tilt, and knee flexion in accordance with the grade of malalignment, to maintain a standing posture with a horizontal gaze [2–10] The greater the malalignment, the more muscle activity is required to maintain a standing posture, leading to fatigue and pain in the back and lower limbs and subsequent deterioration of health-related quality of life. The grade of compensation required is a key factor in evaluating and determining the treatment for degenerative and/or deformative diseases [11]. Conventional global sagittal alignment parameters, such as the sagittal vertical axis [12], T1 pelvic angle (TPA) [13], spino-sacral angle [14], and full balance integrated index [6], are useful, but it is difficult to determine whole body standing alignment (WBSA), which is always compensated based on the cone of economy [1, 15]. Hence evaluation of not only spinal alignment but also WBSA is necessary to elucidate spinal pathology with or without compensation. A recent advance in radiologic devices was realized to show the WBSA using biplanar slot-scanning stereoradiography (EOS®, EOS Imaging, Paris, France), which is becoming the gold standard for evaluating standing alignment [16].

**Fig. 1:**
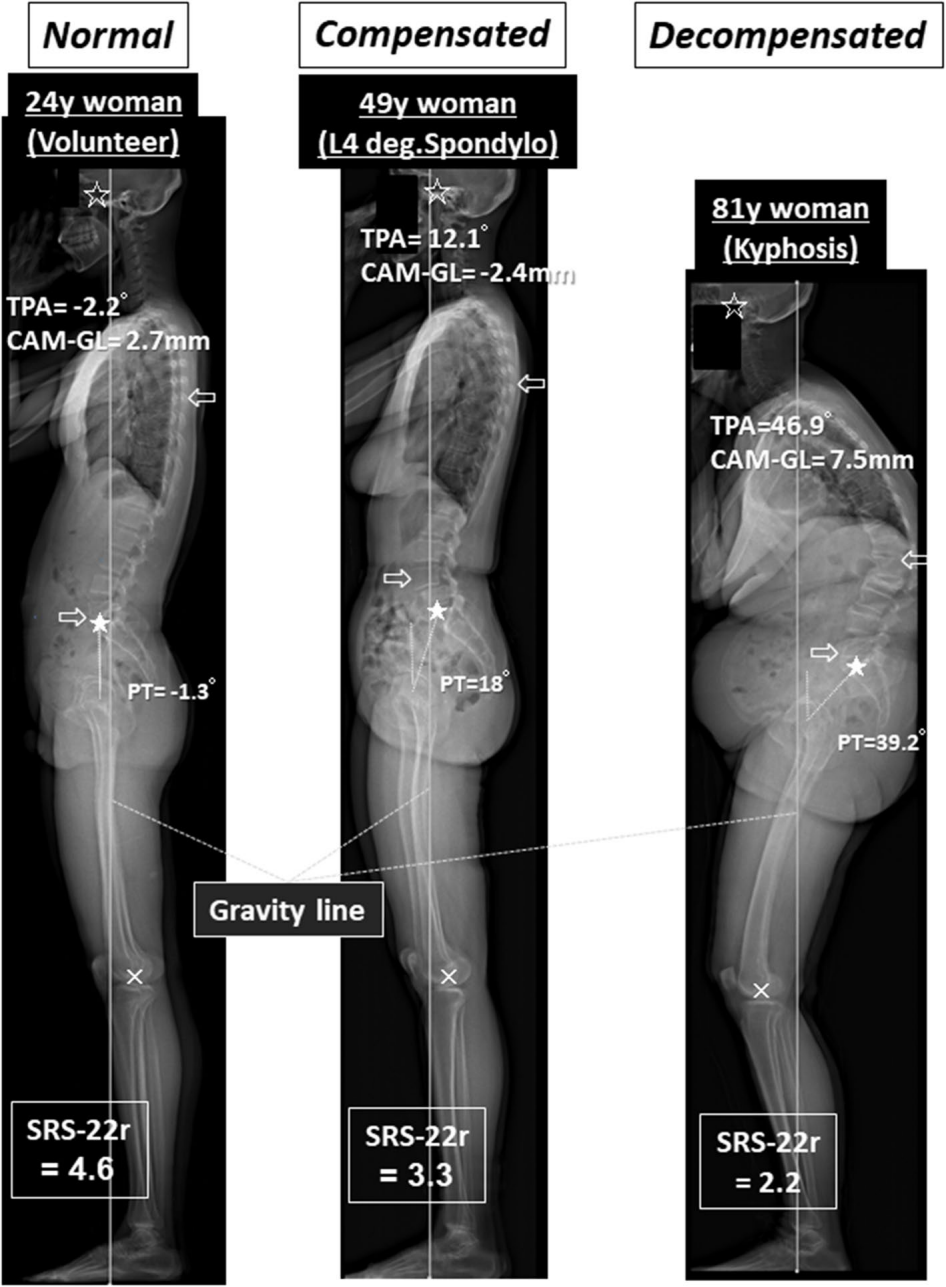
Three stages of compensation [11] in terms of standing whole body sagittal alignment in reference to the gravity line (GL) determined by simultaneous force plate measurement [7] ☆: Center of the acoustic meati (CAM), ⇦: thoracic apex, ⇨: lumbar apex, ★: center of sacral base, ×: center of the knee joints, SRS-22r: the Scoliosis Research Society-22r questionnaire [25, 26]

Several clinical studies of the spinal alignment in reference to the gravity line (GL) have been performed using barycentremetry and/or force plate analyses [17–23], verifying the clinical importance of the GL. We established a WBSA measurement system with EOS images and simultaneous GL capturing by force plate measurement, allowing for accurate WBSA assessment in reference to the GL, the biomechanical datum line [7, 24]. The axial skeleton, from the cranium to the feet, functions as a binding structure [1], and combining the alignment data for these bones serves as the baseline for analyzing the alignment and compensation grade of the standing posture [11]. (Fig. 1).

## Purpose

The objective of this study is to introduce the gravity center (GC) estimation for determining the GL using EOS-based virtual barycentremetry (avatar); to verify its repeatability, reproducibility, and accuracy when compared to estimation by force plate and report preliminary clinical results.

## Methods

### Estimation of the GC

To estimate GC, a research prototype (research software developed by EOS Imaging, not commercialized) was developed to generate patient avatars with density integration by following three steps.

1. 3 D reconstruction of standing whole body skeletons

**Fig. 2:**
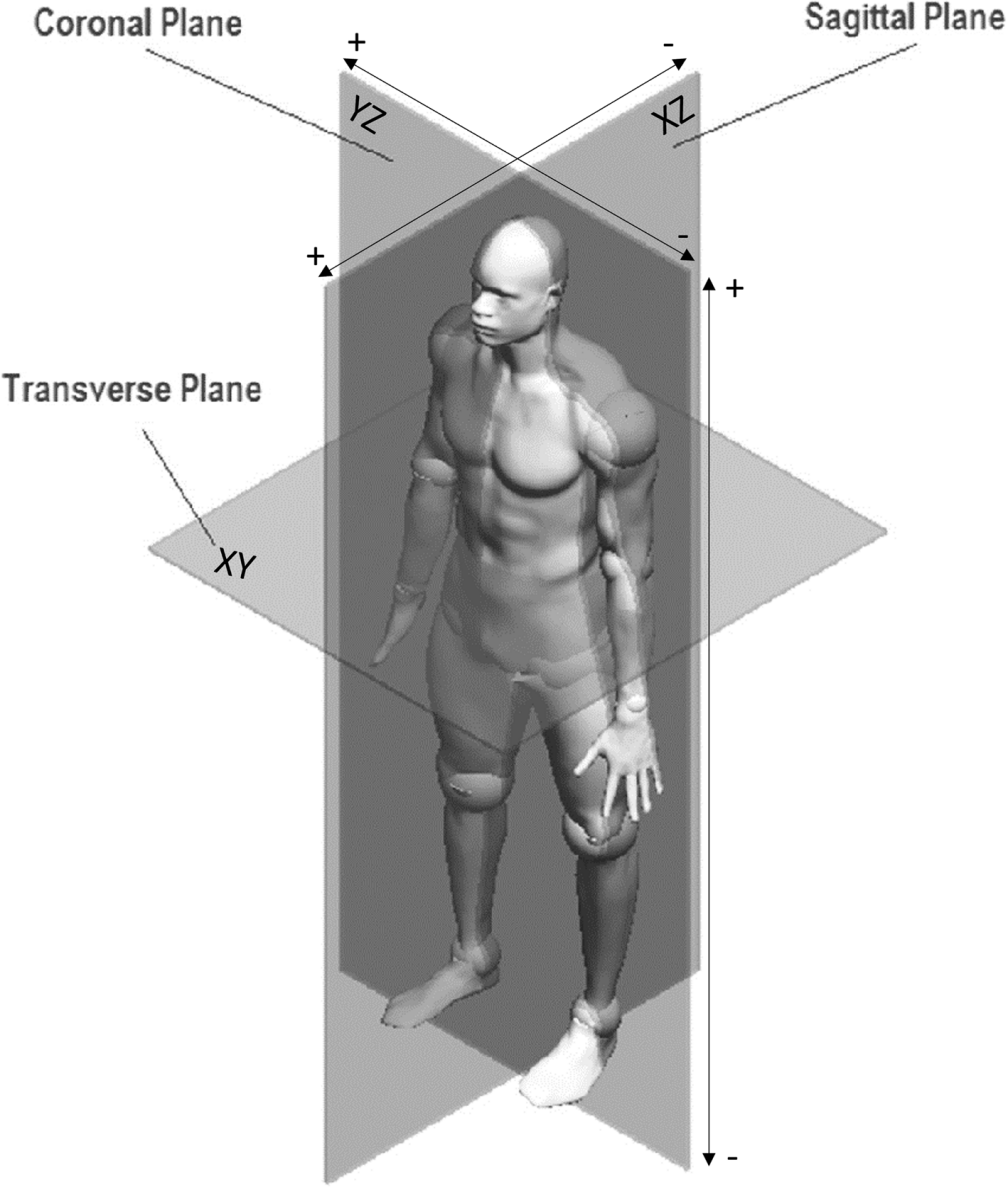
Definition of the 3-dimensional frame. “adapted from Önen, Ü., Botsalı, F. M., Kalyoncu, M., Şahin, Y., & Tınkır, M. (2017). Design and motion control of a lower limb robotic exoskeleton. Design, control and applications of mechatronic systems in engineering, 135-152.”

The prototype uses EOS stereoradiographic full body X-rays and their associated 3-dimensional (3D) model of the axial skeleton obtained with ster EOS® software (EOS Imaging) as inputs [27–29]. The orientation of all the musculoskeletal tissues is defined within the 3D coordinate system (Fig. 2).

2) 3D rasterization of the full body

**Fig. 3:**
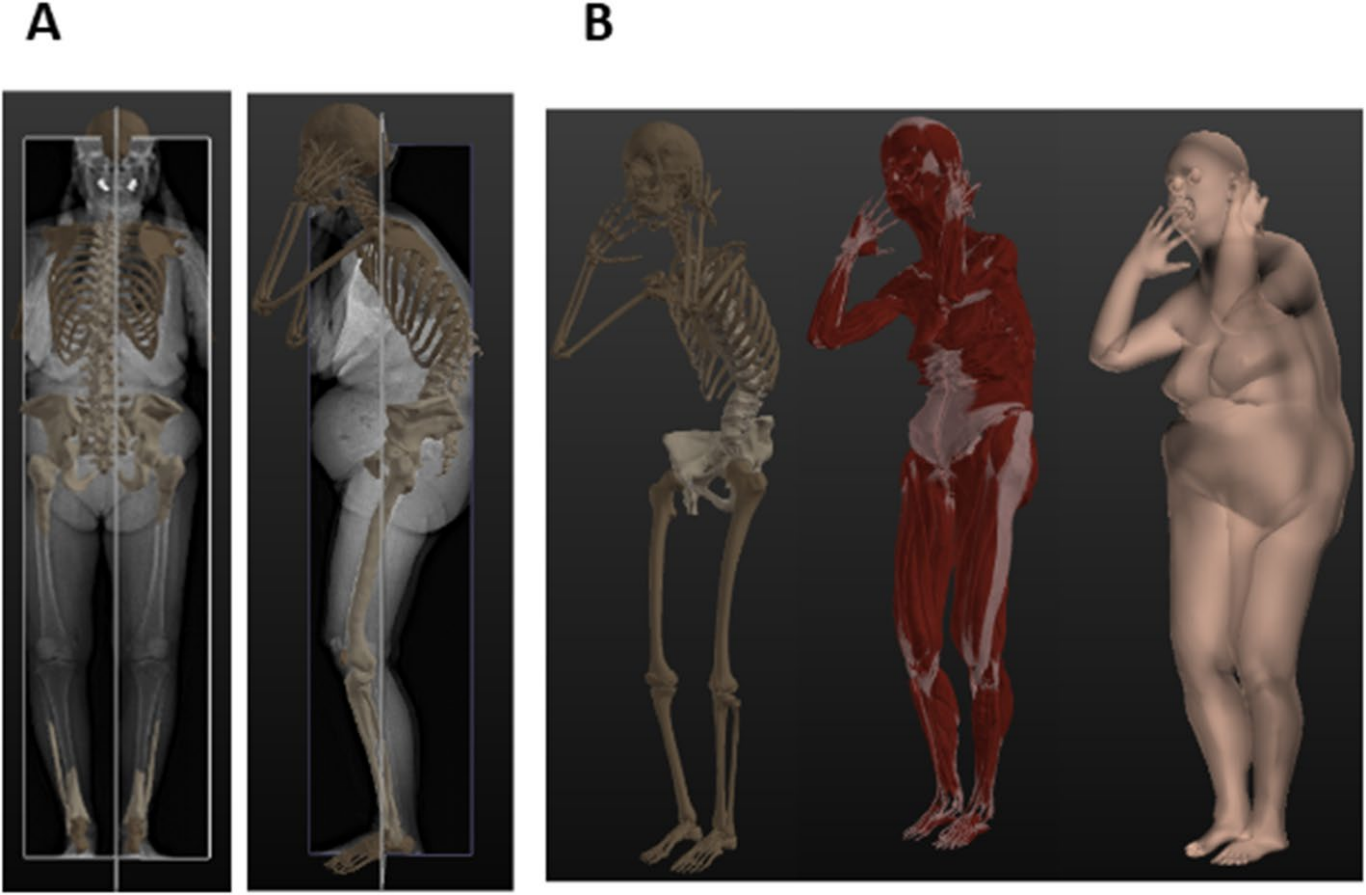
Avatar modeling based on biplanar slot-scanning stereoradiography (EOS®), **A**: 3D modeling of axial skeleton and soft tissue, **B**: Avatar generated by “anatomy transfer”: Bones (left), muscles (center), and skin (right) layers

A reference statistical model from MakeHuman [30] is used to generate, from the information present in the digital imaging and communications in medicine (DICOM) fields (height, weight, sex, age) a morphotype of the patient after the “registration” phase (Fig. 3). The reference model contains all the bony structures as well as the large intra-cavity organs and soft tissues (i.e., skin, fat, muscles, ligaments, etc). Any internal organs that cannot inferred from the medical images of the patient because of invisibility, are interpolated from the reference model: the organ is interpolated based on deformation of bones and skin of the reference model that has been done to match 3D reconstruction based on EOS images of bone and skin. Note that for this study, the reference anatomy is androgenous and does not include sex organs such as ovaries, mammary glands, testicles, and penis, as their small mass should not noticeably bias the barycentremetry (Fig. 3B). The “registration” phase consists in deforming the reference morphology to generate a patient-specific avatar matching the patient data using both EOS X-rays (particularly external soft tissue contours) and EOS based 3D bone reconstructions via a complex constrained optimization called “anatomy transfer” (Anatoscope, Montpellier, France) [31].

The main limitation of the actual registration stage is the inability to accurately fit the patient’s arms and head. Arms can mask the torso of the patient, while the head can be cropped out of the X-ray images. The avatar is positioned with the hands on the cheeks in free-standing position, which is the standard arm position in EOS imaging. This research prototype automatically generated a 3D avatar of the patient that can be displayed graphically in 3D and provides an estimate of the patient’s weight and GC coordinates based on barycentremetry, i.e., an estimation of the application points of the gravity supported by each vertebral segment [32].

**Fig. 4:**
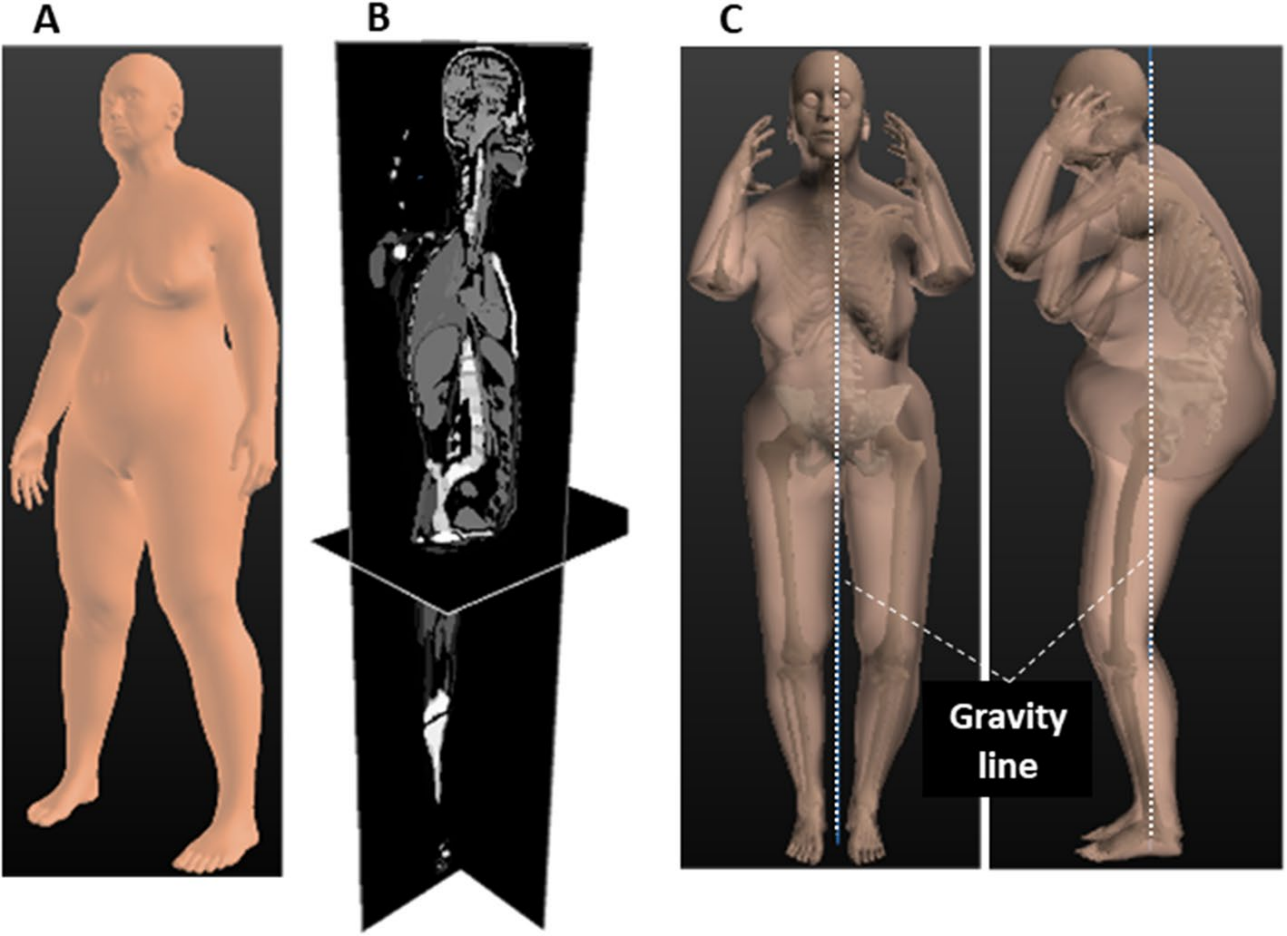
Integration of organ density for computing the global center of gravity in avatar modelling in a clinical case (81-year-old woman, body weight 58 kg, body height 140 cm). **A**: Naked morphotype, **B**: Avatar rasterization: one density per material type, C: Projection of the gravity line (white dotted line) estimated by Avatar

3) Density integrations
  When the registration phase is completed, material densities can then be integrated for every organ volume to compute the weight of each body part. To do so, a 3D rasterization of the full body is performed for 1-mm^3^ voxels in which each voxel comprises a homogeneous material (Fig. 4A and B). The density of each material for each unit volume provides the weight of each voxel, from which the mass and center of mass can be calculated for any sub-part of the body. It is then possible to compute and project onto the floor the GC of the full body (Fig. 4C). As human body material densities can vary between individuals, the density of each material has been optimized to fit the real weight of dozens of patients while remaining in the range of valid densities.

### Participants (Table 1)

All the participants provided written informed consent to participate in the present study and completed all questions on the medical history as well as SRS-22r questionnaire.

**Table 1:** Demographic characteristics of subjects

	Normal (*n* = 10)		Degenerative (n = 10)		Deformative (n = 10)	
	mean ± SE (95% CI)	***p***-value	mean ± SE (95% CI)	***p***-value	mean ± SE (95% CI)	
Parameter		vs. Degenerative		vs. Deformative		ANOVA ***p***-value
		vs. Deformative				
Age	40.1 ± 5.2 (28.3/51.9)		56.6 ± 3.7 (48.2/65.0)		51.4 ± 7.7 (34.0/68.8)	0.1378
Sex (men/women) ^#1^	5/5		4/6		5/5	0.8747 (X^2^)
Body height (cm)	164.3 ± 2.4 (158.8/169.8)		158.0 ± 1.6 (154.3/161.7)		160.9 ± 4.3 (151.3/170.5)	0.3395
Body weight (kg)	60.7 ± 1.5 (55.0/66.4)		60.1 ± 4.1 (50.8/69.4)		62.0 ± 3.4 (54.4/69.6)	0.9216
BMI (kg/cm^2^)	22.5 ± 0.9 (20.5/24.4)		24.0 ± 1.5 (20.6/27.5)		24.2 ± 1.4 (21.0/27.3)	0.6040
SRS-22r	4.48 ± 0.08 (4.32/4.66)	0.0048	3.29 ± 0.19 (2.85/3.72)	0.9963	3.28 ± 0.22 (2.89/3.77)	< 0.0001
		0.0080				

*mean* *+* *SE* Mean value ± standard error, *95% CI* 95% confidence intervals, *BMI* body mass index was calculated as the weight in kilograms divided by the square of the height in meters (kg/m^2^), *SRS-22r* Scoliosis Research Society – 22r Outcome Measures [25, 26] #1: Pearson’s chi-square test (X^2^) was used to test for sex difference. All the parameters except sex were compared using analysis of variance analysis followed by a post-hoc test (Tukey-Kramer’s HSD)

The participants of Normal group were enrolled during Jan 2014 and Sept 2015 from the staffs of the author’s (K.H.) institute who had no history of treatment for spinal disease.

The exclusion criteria of Normal group were:

1. Scoliosis Research Society-22r (SRS-22r) [25, 26] < 4.0
2. scoliosis (Cobb angle > 20°)
3. transitional vertebrae including mobile L6 lumbar vertebrae, sacralization (4 lumbar vertebrae or immobile L5 or L6 vertebrae), and/or 11 or 13 thoracic vertebrae

The patients in Degenerative and Deformative groups were included if they consulted the institute (K.H.) for low back pain and/or radicular pain with or without spinal deformity from January 2014 through July 2015.

The exclusion criteria for Degenerative and Deformative groups were:

1. history of surgical treatment for spinal diseases
2. diagnosed with central nervous system disease or dementia
3. general American Society of Anesthesiologists physical status classification ≥3
4. had transitional vertebrae, including mobile L6 lumbar vertebrae, sacralization (4 lumbar vertebrae or immobile L5 or L6 vertebrae), and/or 11 or 13 thoracic vertebrae

Consequently, the study cohorts were determined as healthy volunteers (Normal group, *n* = 10), patients with spinal degenerative diseases (Degenerative group, n = 10), and patients with spinal deformity (Deformative group, n = 10). Degenerative group included 10 patients with either single-level lumbar degenerative spondylolisthesis (*n* = 5) or lumbar canal stenosis (*n* = 4) or discopathy (n = 1). The Deformity group included 10 patients with either idiopathic scoliosis (n = 4) or degenerative kyphoscoliosis (*n* = 3) or degenerative kyphosis (n = 3). Full body EOS imaging and simultaneous force plate measurement [7] were performed for all the participants.

### Analysis parameters

#### Demographic data

Demographic data, age, sex, body height, body weight [BW], body mass index [BMI], and SRS-22r scores, were collected from all the participants, and compared among the study cohorts.

#### Radiologic measurement by EOS® system and simultaneous force plate measurement

Each participant was asked to stand naturally and comfortably on the force plate with their hands placed on their cheeks. This arm position is recommended to avoid overlap of the hand and T1 vertebral body, which is a key vertebra for measuring thoracic kyphosis, T1–T12. A mirror placed at eye level in the inner wall of the imager box helped the participant maintain a horizontal gaze [33, 34]. Radiographs were obtained from the head to the feet. The force plate measurement (ANIMA Corp., Tokyo, Japan) [35] was simultaneously performed during the EOS scanning (30 s). The track of the GC was recorded in the transverse plane, and the mean location of the track was defined as the mean GC. Then a plumb line from the mean GC was defined as the GL. In the previous study, the repeatability of the force plate was evaluated by intraclass correlation coefficient (ICC) and was 0.99 for track length (cm/s) and 0.92 for the root of the mean square-axis [36]. Spinal curves parameters were computed: C2–7 lordosis as the angle between the C2 endplate and the C7 caudal endplate; thoracic kyphosis as the angle between the T1 rostral endplate and the T12 caudal endplate and lumbar lordosis (LL) as the angle between the L1 rostral endplate and the sacral base. Angles characterizing the pelvis morphology and position were also included in the analysis; pelvic incidence (PI) [17], PI-LL: difference between PI and LL [12], sacral slope and pelvic tilt.

In addition, three parameters characterizing full body alignment were computed: CAM-GL as the distance in cm between the center of the acoustic meati (CAM) and GL in the sagittal plane [7]; T1 pelvic angle (TPA) [13] and Knee flexion as the mean of bilateral knee flexion angles between the line from the hip axis to the midpoint of the bilateral notches of the femoral condyles and the line from the notch to the midpoint of distal tibial joint surfaces [7].

#### Reliability of the GC estimation using the avatar

Three well-trained operators reconstructed the avatar and estimated the GC of the whole body in the standing posture using the method described in section I.1. The reconstruction and estimation of the GC were repeated twice by the 3 operators with a 1-week interval. Consequently, the operators performed 180 reconstructions and estimations of GC. Repeatability was defined as the mean difference of the coordinates of the GC between the first and second measurements for each operator. Reproducibility was defined as the mean difference of the coordinates of the GC among the measurement values determined by the 3 operators. The accuracy of the avatar measurement was assessed by comparing the GC coordinates (x, y) and BW (kg) between the avatar and force plate measurements.

#### Statistical analysis

The data were analyzed to evaluate whether each parameter had a normal distribution using the Shapiro-Wilks test. Mean, standard error of the mean (SE), and 95% confidence interval (CI) were calculated. All values for each parameter, except sex, were then compared among the groups using analysis of variance (ANOVA) followed by a post-hoc test (Tukey-Kramer’s HSD). The chi-square test was used to compare sex differences among the groups.

Repeatability and reproducibility were analyzed by paired t-test. Intra-rater and inter-rater ICC of the GC coordinates (x, y) in cm estimated by Avatar was investigated for twice measurements by three operators. The model, 1,k by Shrout and Fleiss was calculated for intra-rater ICC, and the model, 2,k was used for inter-rater ICC [37].

To assess the accuracy of the avatar estimation, the GC coordinates and BW of the avatar were compared to those obtained by force plate estimations using a paired t-test. Differences in the BW measurements among the avatar, force plate, and conventional scale were compared by ANOVA followed by a host-hoc test (Tukey-Kramer’s HSD). These accuracy evaluations were performed for all participants and among the diagnostic groups. Furthermore, Bland-Altman analysis for the difference of GC coordinate (X, Y) between avatar and FP measures was performed to assess the agreement of the two methods.

The JMP software package (ver.9.0.0, SAS Institute, Cary, NC) was used for all statistical analyses. A *p*-value of less than 0.05 was considered statistically significant.

## Results

### Demographic data

Age sex, body height, BW, and BMI were not significantly different among the groups (Table 1). The SRS-22r scores of the Normal group were significantly higher than those of the Degenerative and Deformative groups. The SRS-22r scores did not significantly differ between the Degenerative and Deformative groups (Table 1).

### Radiologic measurement by the EOS system

There was significant difference in TPA, LL, PI-LL, SS, PT, and Knee flexion among the groups, with greater values in TPA, PI-LL, PT, and Knee flexion, and with lesser values in LL and SS, in Degenerative and Deformative compared with Normal group. All the values, however, did not differ between Degenerative and Deformative groups (Table 2).

**Table 2:** Results of radiologic parameters measure by EOS system

	Normal (*n* = 10)		Degenerative (*n* = 10)		Deformative (*n* = 10)	
	mean ± SE (95% CI)	***p***-value	mean ± SE (95% CI)	***p***-value	mean ± SE (95% CI)	
Parameter		vs. Degenerative		vs. Deformative		ANOVA ***p***-value
		vs. Deformative				
CAM-GL (cm)	0.7 ± 0.9 (−1.2/2.5)	0.20	−1.0 ± 0.9 (−2.9/0.8)	0.68	0.6 ± 0.9 (−1.2/2.5)	0.32
		0.98				
TPA (°)	2.7 ± 4.4 (−6.3/11.3)	**< 0.01**	19.5 ± 4.4 (10.5/28.5)	0.89	18.2 ± 4.4 (9.2/27.2)	**< 0.05**
		0.23				
C2–7 lordosis (°)	−3.6 ± 4.7 (−13.2/6.0)	0.23	4.4 ± 4.7 (−5.2/13.9)	0.99	6.3 ± 4.7 (−3.3/15.8)	0.30
		0.66				
TK (°)	44.3 ± 3.8 (36.5/52.2)	0.05	32.3 ± 3.8 (24.4/40.1)	0.98	33.4 ± 3.8 (25.5/41.2)	0.07
		0.34				
LL (°)	61.0 ± 5.1 (50.6/71.4)	**< 0.01**	39.8 ± 5.1 (29.4/50.2)	0.92	35.8 ± 5.1 (25.3/46.2)	**< 0.01**
		**< 0.05**				
PI (°)	49.9 ± 3.4 (42.9/56.9)	0.38	55.0 ± 3.4 (48.0/62.0)	0.34	49.0 ± 3.4 (42.0/56.0)	0.41
		0.99				
PI-LL (°)	−11.1 ± 5.9 (−23.3/1.1)	**< 0.01**	15.2 ± 5.9 (3.0/27.4)	0.98	13.2 ± 5.9 (1.1/25.4)	**< 0.01**
		0.08				
SS (°)	44.4 ± 2.9 (38.5/50.3)	**< 0.05**	31.9 ± 2.9 (26.0/37.8)	0.84	29.8 ± 2.9 (23.9/35.7)	**< 0.01**
		**< 0.05**				
PT (°)	5.5 ± 3.7 (−2.1/13.1)	**< 0.01**	23.1 ± 3.7 (15.5/30.7)	0.68	19.2 ± 3.7 (11.6/26.8)	**< 0.01**
		0.15				
Knee flexion (°)	0.6 ± 2.3 (−4.0/5.2)	1.00	1.0 ± 2.3 (−3.6/5.6)	0.20	8.5 ± 2.3 (3.8/13.1)	**< 0.05**
		0.18				

*Mean ± SD* Mean value ± standard error, *95% CI* 95% confidence intervals, *CAM-GL* Offset between the center of the acoustic meati (CAM) and gravity line (GL) using the force plate measurement, *TPA: T1* pelvic angle. Sum of T1 inclination on the hip axis and pelvic tilt (PT) [13], *TK* thoracic kyphosis between T1 cranial endplate and T12 caudal endplate. *LL* lumbar lordosis between L1 cranial endplate and base of sacrum, PI: pelvic incidence, *SS* sacral slope. All the parameters except sex were compared using analysis of variance analysis followed by a post-hoc test (Tukey-Kramer’s HSD)

### Repeatability and reproducibility of the avatar reconstruction and measurement

Repeatability was ≤0.07 cm for both the x and y coordinates and ≤ 0.29 kg for BW. Reproducibility was - ≤ 0.02 cm for both the x and y coordinates and ≤ 0.28 kg for BW (Table 3). Mean intra-rater ICC values of the GC coordinates (x, y) of the 3 operators were (1.000, 0.999), (0.999, 0.999), and (0.999, 0.999), respectively. Mean inter-rater ICC value of the GC coordinates (x, y) among the 3 operators was (1.000, 0.999) (Table 4).

**Table 3:** Repeatability and reproducibility of the estimation of the coordinates (x, y) in cm of GC and BW in kg

Parameters		Repeatability	Reproducibility
**All participants**	**x**	0.06	0.02
	**y**	0.05	0.02
	**BW**	0.23	0.18
**Normal**	**x**	0.04	0.01
	**y**	0.03	0.01
	**BW**	0.13	0.06
**Degenerative**	**x**	0.07	0.02
	**y**	0.04	0.01
	**BW**	0.23	0.13
**Deformative**	**x**	0.06	0.02
	**y**	0.07	0.02
	**BW**	0.29	0.28

Repeatability was defined as the mean difference in value between the first and second measurements of 3 operators. Reproducibility was defined as the mean difference among the measurement values of the 3 operators

**Table 4:** Intra-rater ICC and Inter-rater ICC of the GC coordinates (x, y) estimated by Avatar for all the participants (*n* = 30) [37]

		Intra-rater ICC	Inter-rater ICC
**operator 1**	**x**	1.000 (0.999–1.000	
	**y**	0.999 (0.997–0.999)	
**operator 2**	**x**	0.999 (0.999–1.000)	
	**y**	0.999 (0.999–1.000)	
**operator 3**	**x**	0.999 (0.999–1.000)	
	**y**	0.999 (0.998–1.000)	
**x**			1.000 (0.999–1.000)
**y**			0.999 (0.999–1.000)

The model, 1,k by Shrout and Fleiss was calculated for intra-rater ICC, and the model, 2,k was used for inter-rater ICC [37]. The values are shown as mean (95% confidence interval)

### Accuracy of the avatar reconstruction and measurement

Comparison between the avatar and force plate measurements

1. Estimation of GC coordinates and BW

None of the parameters examined, except Y-coordinate values, differed significantly between the avatar and force plate measurements in any of the participants (paired t-test) and among subgroups (ANOVA followed by Tukey-Kramer’s HSD analysis) (Table 5).

**Table 5:** Coordinates (x, y) in cm of the gravity center and the body weight in kg among groups

***Parameter***			***Normal****(n = 10)*	***Degenerative****(n = 10)*	***Deformative****(n = 10)*
Coordinate	x (cm)	*Avatar*	−0.19 ± 0.42 (−1.15/0.77)^*1^	−1.55 ± 0.31 (−2.25/−0.85) ^*2^	−0.32 ± 0.24 (− 0.86/0.23) ^*3^
		*Force plate*	− 0.22 ± 0.43 (−1.21/0.77)	−1.69 ± 0.31 (− 2.4/− 0.99)	−1.29 ± 0.46 (− 2.33/− 0.24)
	y (cm)	*Avatar*	0.09 ± 0.22 (− 0.4/0.58) ^*4^	0.26 ± 0.23 (− 0.25/0.78) ^*5^	0.36 ± 0.30 (− 0.31/1.04) ^*6^
		*Force plate*	− 0.16 ± 0.19 (− 0.6/0.28)	−0.01 ± 0.30(− 0.68/0.65)	0.05 ± 0.31 (− 0.65/0.76)
BW (kg)		*Avatar*	62.1 ± 2.5 (56.4/67.7) ^*7^	61.6 ± 3.9 (52.6/70.5) ^*8^	63.2 ± 3.3 (55.7/70.7) ^*9^
		*Force plate*	61.1 ± 2.3 (55.9/66.3)	60.8 ± 4.2 (51.4/70.2)	62.0 ± 3.5 (54.1/69.9)
		Scales	60.7 ± 2.5 (55.0/66.4)	60.7 ± 4.0 (51.7/69.7)	61.9 ± 3.4 (54.2/69.6)

Mean value ± standard error, lower / upper 95% confidence intervals, *BW* body weight

Coordinate (X, Y) was compared by paired *t*-test, and BW was compared by ANOVA in each group

*1: v.s. Force plate, *p* = 0.7170, *2: v.s. Force plate, *p* = 0.6850, *3: v.s. Force plate, *p* = 0.555, *4: v.s. Force plate, *p* = 0.0002, *5: v.s. Force plate, *p* = 0.0136, *6: v.s. Force plate, *p* = 0.0024, *7: v.s. Force plate and Scales, *p* = 0.9177, *8: v.s. Force plate and Scales, *p* = 0.9864, *9: v.s. Force plate and Scales, *p* = 0.9590

2) Difference in the GC estimated by the avatar and force plate among groups

**Fig. 5:**
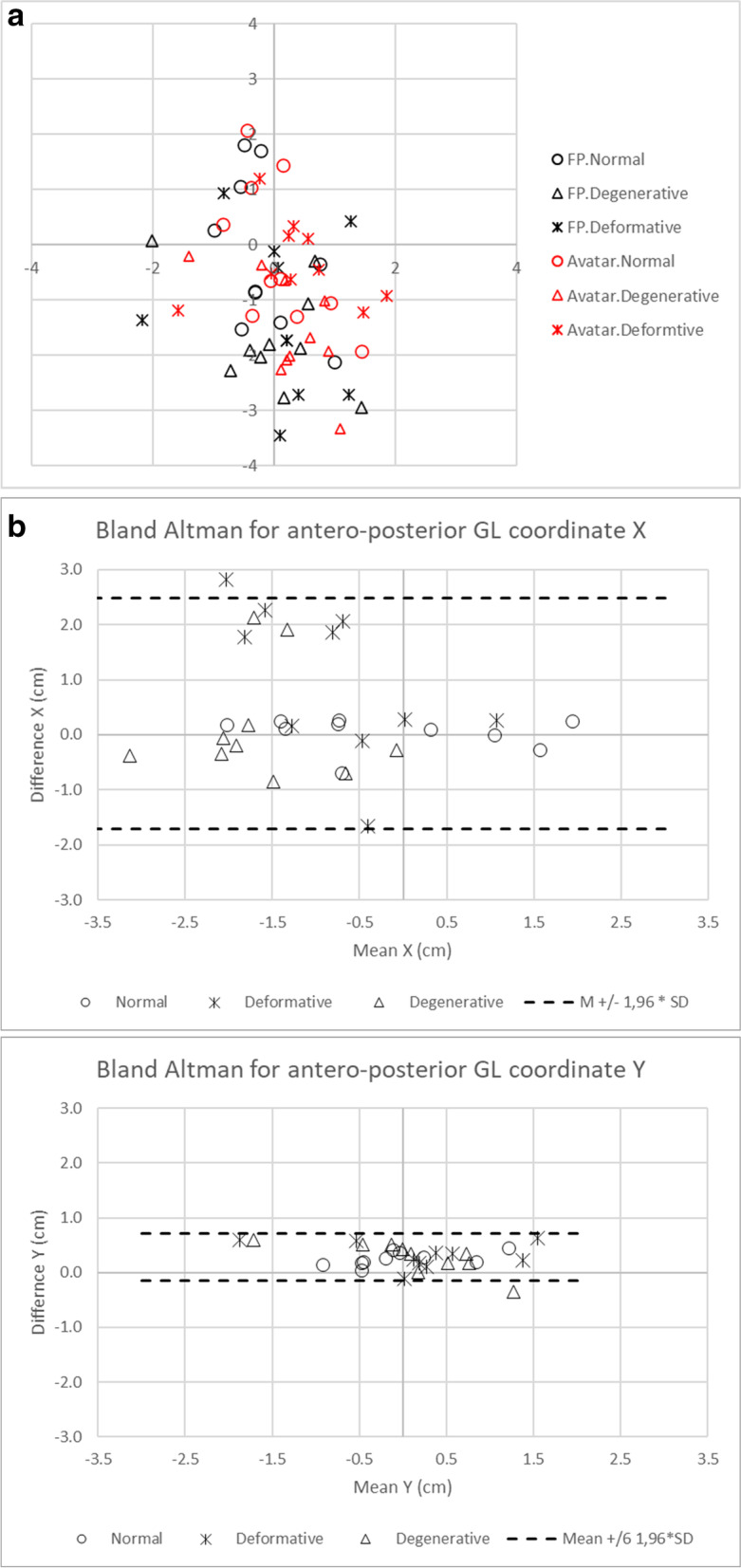
**A** Scattergram of all gravity center locations estimated by avatar (Avatar, red markers) and force plate (FP, black markers) measurement divided by groups (Normal, Degenerative, Deformative). **B** Bland-Altman plots for the difference of GC coordinate (X, Y) between avatar and FP measures. Each marker is shown as the value of Avatar minus FP measurements divided by the mean value. Limits of agreement in black dashed lines correspond to mean of the difference plus or minus 1.96 times the standard deviation of the difference

All the plots of the GC are located within 4 m in X-coordinate, anteroposterior direction, and within 3 cm in Y-coordinate, lateral direction in both Avatar and FP measurements (Fig. 5A).

The mean of absolute difference of the GC coordinates (mean + standard error [SE], cm), Avatar minus force plate measurement, in X-axis, i.d. anteroposterior direction gradually increased in the order of Normal (0.23 + 0.23), Degenerative (0.71 + 0.23), and Deformative (1.32 + 0.23) groups (*p* = 0.0093), whereas those of Y-axis, i.e. lateral direction, showed no significant differences (*p* = 0.3906) (Table 6). Bland-Altman plots for the difference of GC coordinates (X, Y) between avatar and FP measurements revealed the widely scattered plots in X-coordinate in which all but one deformative patient were within the limit of + 1.96*standard deviation, and the slightly scattered plots in Y-coordinate in which all but one degenerative group were within the limit of + 1.96*standard deviation (Fig. 5B).

**Table 6:** Absolute difference in the coordinate (x, y) of the GC and BW between the avatar and force plate measurements

		Coordinate of gravity center (cm)		Body weight (kg)
		x	y	
**All participants**	Mean ± SD	0.75 ± 0.15	0.31 ± 0.03	1.56 ± 0.18
	Minimum; maximum	0.01; 2.82	0.01; 0.63	0.04; 3.49
**Normal**	Mean ± SD	0.23 ± 0.23	0.25 ± 0.06	1.30 ± 0.35
	Minimum; maximum	0.01; 0.70	0.04; 0.44	0.04; 3.34
**Degenerative**	Mean ± SD	0.71 ± 0.23	0.35 ± 0.06	1.72 ± 0.27
	Minimum; maximum	0.06; 2.12	0.01; 0.60	0.18; 2.86
**Deformative**	Mean ± SD	1.32 ± 0.23	0.34 ± 0.06	1.66 ± 0.34
	Minimum; maximum	0.11; 2.82	0.11; 0.63	0.14; 3.49

Mean ± standard error (SE), Maximum / minimum. x and y show the coordinates of the gravity center (x, y)

## Discussion

Humans are characterized by bipedalism, which was realized by developing cervical and lumbar lordosis with pelvic adaptation between the spine and lower limbs to maintain a horizontal gaze and to free the upper limbs. In the standing posture in which the force by body weight are equal to ground reaction forces, or such that none surpasses the sum of the others, the center of mass should project as closely as possible toward the center of a reduced polygon situated between the 2 ft in a stable position and in an area requiring only small constant rebalancing effort. If the center of mass tends to project outside this polygon, the rebalancing efforts to compensate the malaligned parts become much more important [1, 38].

The compensation stages for standing posture, normal, compensated, and decompensated, correlate with the rebalancing effort and also health-related quality of life measurements. The threshold values of key alignment parameters for severe disability (decompensated stage) are T1 pelvic angle > 30°, C2 to C7 lordosis > 13°, pelvic incidence-lumbar lordosis > 30°, pelvic tilt > 28°, and knee flexion > 8° [11]. Obtaining these thresholds is valuable, but measuring the parameters takes time and is inconvenient for daily clinical practice. Once we can visualize the WBSA in reference to the GL, we can easily qualitatively evaluate the WBSA and the compensation stage (Fig. 1). For example, in normal to compensated stages, CAM and the sacral base is just on the GL, the apex of the thoracic kyphosis (near T7) is approximately 5.0 cm posterior to GL, the apex of the lumbar lordosis (near L4) is approximately 0.6 cm anterior, the hip axis is approximately 1.4 cm anterior, the knee joint is approximately 2.4 cm posterior, and the ankle joint is approximately 4.8 cm posterior to the GL [24]. Given the fundamental alignment of the key parts of the axial skeleton, it is easy to judge the grade of compensation in terms of CAM, thoracic–lumbar vertebrae, pelvis, and lower limbs (Fig. 1).

In the present study, we established a new GL estimation method using a virtual avatar based on a 3D skeleton obtained from EOS imaging, in addition to whole body bone alignment measurements made on this 3D skeleton, making it possible to quickly grade the compensation stage. The GL estimation required no additional tools during the EOS® full body biplanar acquisition and lasts around 5 min for each patient, future automation is currently under evaluation. Repeatability of the GC estimation by 3 operators using the avatar in the healthy volunteers and patients with degenerative and deformative lesions was 0.06 cm in the X-axis and 0.05 cm in the Y-axis, and that of BW was 0.23 kg, respectively. Repeatability, defined as the mean difference between the first and second measurements of the 3 operators, in all subgroups was less than 0.1 cm for the GC coordinate estimation and less than 0.3 kg for BW (Table 3). The mean intra-ICC of the GC coordinates of the 3 operators was ≥0.999 and the mean inter-ICC of the parameters among the 3 operators was ≥0.999. The coordinate values were not significantly different between the avatar and force plate measurements in any subgroup (Table 4). All the plots of the GC in participants are located within 4 m in X-coordinate and within 3 cm in Y-coordinate in both Avatar and FP measurements (Fig. 5A). The mean GC difference (Avatar minus force plate measurement) in the X-axis gradually increased in the order of the Normal (0.23 cm), Degenerative (0.71 cm), and Deformative (1.32 cm) groups, whereas those of Y-axis showed no significant differences (Table 6). The difference of GC coordinates in X-coordinate was greater than that in Y-coordinate. The values were, however, all but one participant were within the limit of + 1.96*standard deviation in both coordinates (Fig. 5B). These results on the repeatability, reproducibility, and accuracy demonstrate the high reliability of the avatar measurement.

This suggests that most of the participants were in the normal or compensated stages. The mean difference in distance between the GC coordinates in the avatar and force plate measurements, however, were significantly different in the order of the Normal (0.37 cm), Degenerative (0.87 cm), and Deformative (1.44 cm) groups in sagittal plane. By comparison, the accuracy of a laboratory-grade force plate is approximately 0.5 cm [23] and 1 cm for the widely available force plate, the Wii Balance Board (Nintendo, Kyoto, Japan) [39]. There were 8 outliers for whom the difference of the coordinates between the avatar and force plate measurement was greater than 1 cm (Fig. 5B). Among the 8 outliers, 6 were in the Deformative group and 2 were in the Degenerative group. Therefore, the GL estimated using the avatar tended to be less accurate for patients with degenerative or deformative lesions than for participants without lesions. This is a limitation in accuracy of the present study due to the heterogenous disease groups especially for the patient with spinal deformity.

Possible reasons for the difference in the GL estimation between the avatar and force plate measurements in X-axis, i.d. sagittal plane, (mean + SE, cm): Normal (0.23 + 0.23), Degenerative (0.71 + 0.23), and Deformative (1.32 + 0.23) groups (*p* = 0.0093) (Table 6) are: 1) The generic avatar position of the head and arms, as this is not patient-specific, could generate some errors. 2) Some patients can have body parts outside the field of view of the EOS images, therefore the avatar body contours estimation might not be accurate in this situation. 3) Lastly, for patients having a large postural sway, the image acquired by the EOS system can lead to a GL estimation different from the mean of the force plate values recorded during acquisition.

These imperfect images tend to especially occur in participants with deformative spine lesions. Haddas et al. [15] investigated body sway during Romberg’s test using a full body marker set and a human motion capture system, calculating the 3D cone of economy dimension and range of sway of the head and center of mass. The investigation revealed that adult deformity patients have larger cone of economy dimensions and increased sway compared with non-scoliotic controls [15]. This supports the third possible reason above. The GL estimation method, however, is accurate once equipped in the EOS software, contributing to improve the diagnostic ability in WBSA from a biomechanical point of view in daily clinical practice. The authors consider that the advantage outweighs the limitations.

## Conclusions

Gravity center, which delivers the GL (the datum line), reinforces evaluation of WBSA. In this study, we introduced a new GC estimation method using EOS®-based virtual barycentremetry (avatar), and investigated the repeatability, reproducibility, and accuracy of the measurement system with preliminary clinical results for participants with normal, degenerative, and deformative spines.

Repeatability and reproducibility of the avatar was high with intra-ICC and inter-ICC values ≥0.999. There were no significant differences in the coordinate values and BW estimation between the avatar and force plate measurements, demonstrating the high accuracy of the method.

The new GC estimation in addition to WBSA measurement is considered reliable and accurate. This could provide a rapid qualitative evaluation method for patients with various spinal malalignment pathologies.

## Data Availability

The datasets used and/or analysed during the current study are available from the corresponding author on reasonable request.

## Abbreviations

3D: 3-dimensional
ANOVA: Analysis of variance
Avatar: EOS®-based virtual barycentremetry
BW: Body weight
BMI: Body mass index
CAM: Acoustic meati
CI: Confidence interval
DICOM: Digital imaging and communications in medicine
EOS®: Biplanar slot-scanning stereoradiography
GL: Gravity line
GC: Gravity center
intra-ICC: Intraclass correlation coefficient
LL: Lumbar lordosis
PI: Pelvic incidence
TPA: T1 pelvic angle
WBSA: Whole body standing alignment
